# ANT1 suppression inhibits the progression of colorectal cancer by suppressing PINK1/Parkin-mediated mitophagy: ANT1 suppression inhibits CRC via mitophagy

**DOI:** 10.3724/abbs.2025154

**Published:** 2025-11-03

**Authors:** Jin Ji, Mingrui Jiang, Shantanu Baral, Qiannan Sun, Dong Tang, Wei Wang, Jun Ren, Daorong Wang

**Affiliations:** 1 Northern Jiangsu People’s Hospital Affiliated to Yangzhou University Yangzhou 225001 China; 2 General Surgery Institute of Yangzhou Yangzhou University Yangzhou 225001 China; 3 Yangzhou Key Laboratory of Basic and Clinical Transformation of Digestive and Metabolic Diseases Yangzhou 225001 China; 4 Medical Experimental Research Center Northern Jiangsu People’s Hospital Yangzhou 225001 China

**Keywords:** adenine nucleotide translocator 1, colorectal cancer, mitophagy, Parkin, PINK1

## Abstract

Mitochondrial dysfunction is closely related to tumor development. Adenine nucleotide translocator 1 (ANT1), which promotes ADP/ATP translocation across the inner mitochondrial membrane, is an important protein involved in mitochondrial function and plays a role in a variety of diseases, including cancers. However, its role in colorectal cancer (CRC) progression remains poorly understood. This study aims to explore the potential role of ANT1 in CRC and its relationship with mitophagy. Through immunohistochemical analysis, we find that ANT1 expression is significantly higher in the tumor tissues of CRC patients than in adjacent normal tissues and that its overexpression is associated with poor prognosis. Further experiments demonstrate that *ANT1* knockdown significantly inhibits CRC cell proliferation, migration, and invasion and leads to mitochondrial dysfunction, increased ROS production, and apoptosis by suppressing mitophagy. Mechanistically, *ANT1* knockdown downregulates the PINK1/Parkin pathway, thereby inhibiting mitophagy activity. Notably, PINK1 overexpression partially rescues the cellular dysfunction induced by *ANT1* knockdown, suggesting a potential role for PINK1 in reversing the suppression of mitophagy. *In vivo* xenograft models also show that *ANT1* knockdown markedly inhibits tumor growth. In conclusion, ANT1 may play a critical role in CRC progression by regulating mitophagy, providing a basis for its potential as a therapeutic target.

## Introduction

Colorectal cancer (CRC) is the third most common cancer and the third leading cause of cancer-related death [ 1, 2] . Studies estimate that by the year 2040, the burden of CRC is projected to increase to 3.2 million new cases and 1.6 million deaths, with the majority of cases occurring in countries with high or very high human development indices [ 3– 5] . Recent research has shown a steady increase in the incidence of CRC among young individuals [ 6, 7] . Surgery is the treatment of choice in the early stages, but patients with advanced disease have lower survival rates and rely on neoadjuvant chemotherapy and targeted immunotherapies [ 8, 9] . Currently, some progress has been made in the development of targeted drugs for the treatment of CRC, but their therapeutic effects are affected by the location and molecular characteristics of the tumor [ 10, 11] . Therefore, more scientific and effective treatments for CRC are becoming a major challenge in our new era of precision medicine.

Mitochondria, commonly known as the cellular “powerhouse” are critical for maintaining homeostasis and supporting diverse cellular functions while also playing an important role in determining cell fate by participating in programmed cell death [ 12– 14] . Adenine nucleotide translocase (ANT), a dual-function protein located on the inner membrane of mitochondria, regulates adenosine triphosphate (ATP)/adenosine diphosphate (ADP) exchange for metabolism and is part of the mitochondrial permeability transition pore, which regulates cell death [ 15– 19] . The ANT family consists of four subtypes, ANT1, ANT2, ANT3, and ANT4, which are encoded by four nuclear genes whose expression is tightly regulated. ANT1, encoded by the nuclear gene *SLC25A4*, is a heart- and muscle-specific subtype of the ANT mitochondrial inner membrane protein family [20]. Hoshino *et al*. [21] reported that ANT1 is essential for mitophagy, as its knockout in mice leads to reduced mitophagy and the accumulation of abnormal mitochondria.

Mitophagy is a critical mitochondrial quality control mechanism that eliminates damaged mitochondria and reduces the production of reactive oxygen species (ROS) [22]. Changes in mitochondrial function play pivotal roles in cancer development, as mitophagy can either support cell survival or promote cell death depending on the cellular environment, functioning as a double-edged sword in tumor cells [23]. On one hand, mitochondria are crucial for energy production in eukaryotic cells, and their maintenance is essential for cell survival [24]. Mitophagy helps alleviate stress by clearing dysfunctional mitochondria [25]. On the other hand, mitophagy suppresses tumor growth by removing defective mitochondria, which might otherwise transform cells and contribute to tumorigenesis [26].

Since ANT1 is currently less reported in CRC, we initially examined the expression of ANT1 in CRC and its association with the clinicopathological characteristics of patients and analyzed its prognostic value in CRC patients. We subsequently investigated whether mitophagy serves as a key mechanism for the involvement of ANT1 in CRC, aiming to provide novel mechanistic insights into the role of ANT1 in the development and progression of CRC.

## Materials and Methods

### Clinical data and samples

A total of 48 specimens were collected from patients who underwent CRC surgery at Northern Jiangsu People’s Hospital from July 2016 to June 2017. Each sample included cancer tissue and matched cancer-adjacent tissue located more than 6 cm away from the tumor margin. Preoperative neoadjuvant chemotherapy was not administered to any of the patients, and postoperatively, all patients were confirmed histopathologically to have malignant tumors, with secondary metastasis ruled out on the basis of imaging and intraoperative findings. The acquisition of tissue samples and clinical data was conducted with informed consent from the patients and their families. The study was conducted in accordance with the Declaration of Helsinki and was approved by the Medical Ethics Committee of Northern Jiangsu People’s Hospital (approval No. 2020KY-137). Sex, age, tumor location, maximum tumor diameter, tumor differentiation, pathological type, stage, and number of lymph node metastases were recorded, and regular telephone follow-ups were conducted. The total follow-up period was 60 months, during which the patient’s health status and recurrence were recorded.

### ANT1 immunohistochemistry and expression score

Immunohistochemistry (IHC) was performed on 48 CRC tissue samples and adjacent non-cancerous tissues. Paraffin-embedded tumor tissues were sectioned at a thickness of 4 μm, deparaffinized, rehydrated, and subjected to citrate buffer antigen retrieval, followed by blocking, incubation with SLC25A4 rabbit pAb (861244; ZEN Bio, Chengdu, China) overnight at 4°C and HRP-conjugated anti-rabbit IgG (SV0002; Boster, Wuhan, China), DAB visualization, hematoxylin counterstaining, dehydration, and mounting. After staining, the slides were evaluated by three researchers who were unaware of the sample origins. For evaluation, six random fields were chosen for each slide. The researchers scored the proportion of positively stained cells (0–3) and the intensity of staining (0–3). The scoring criteria for the proportion of stained cells were as follows: ≤ 25% positively stained cells were scored as 0, 26%–50% as 1, 51%–75% as 2, and > 75% as 3. The scoring criteria for staining intensity were as follows: no staining in the field, 0 point; faint yellow, 1 point; light brown, 2 points; and dark brown, 3 points. The total score of immunohistochemical staining was calculated by multiplying the proportion of positively stained cells by the staining intensity. A total score ≤ 4 was considered low expression of ANT1, whereas a total score > 4 was considered high expression of ANT1.

### Cell culture

The human normal colon epithelial cell line NCM460; the human colon cancer cell lines LOVO, HT29, and HCT-116; and the human colorectal adenocarcinoma cell lines SW480 and SW620 were obtained from iCell Bioscience, Inc. (Shanghai, |China). All the cells were cultured in Dulbecco’s modified Eagle’s medium (DMEM) containing 10% fetal bovine serum and 100 units/mL penicillin and streptomycin at 37°C in a humidified incubator with 5% CO _2_.

### Lentiviral transfection

Twenty-four hours before transfection, the cells were seeded into corresponding 6-well plates at a density of 1 × 10 ^5^ cells/mL, ensuring a cell density of 2 × 10 ^5^ cells/mL at the time of transfection. The original culture medium was replaced by 2 mL of fresh medium containing 6 mg/L polybrene, and 20 μL of lentiviral suspension was added, followed by incubation at 37°C. After 8 h, the transfection efficiency was observed. Fresh culture medium (2 mL/well) was then added, and the cells were cultured in an incubator at 37°C with 5% CO _2_. Three to four days post-transfection, puromycin was added for stable selection. For lentiviral knockdown, the cells were divided into the following groups: control, empty vector (vector), and *ANT1* knockdown lentivirus (shANT1#1/#2/#3) groups, which were subsequently transfected into SW620 and SW480 cells. The rescue experiment was designed to assess the relationship between ANT1 and PINK1 targeting and included four groups: control, empty vector (vector), *ANT1* knockdown lentivirus + empty vector (shANT1 + oeNC), and *ANT1* knockdown lentivirus + PINK1 overexpression lentivirus (shANT1 + oePINK1). Western blot analysis and qPCR were used to verify the expressions of ANT1 and PINK1 in the transfected cells. Both the *ANT1* knockdown lentivirus and the PINK1 overexpression lentivirus were provided by Genechem (Shanghai, China). Table 1 lists the shRNA sequences used to target human ANT1 complementary DNA.

**Table:** **Table 1** shRNA sequences used in this study

Number	Target sequence (5′→3′)
shANT1#1	GTGTTGTATGATGAGATCAAA
shANT1#2	CCTTTGACACTGTTCGTCGTA
shANT1#3	GACACTGTTCGTCGTAGAATG

### Western blot analysis

After transfection for 72 h, the cells from each group were collected and washed three times with PBS. The cell lysates were added and centrifuged to extract total cellular protein, the concentration of which was determined via a bicinchoninic acid assay kit (Beyotime, Shanghai, China). The proteins were denatured by boiling and then subjected to sodium dodecyl sulfate-polyacrylamide gel electrophoresis for 1–2 h. The proteins were transferred to a membrane for 30–50 min. The membrane was incubated with primary antibodies overnight at 4°C, followed by incubation with HRP conjugated secondary antibodies (BA1050, BA1055; Boster) at room temperature for 1–2 h. Finally, the membrane was exposed to enhanced chemiluminescence exposure solution and imaged. The antibodies used were as follows: anti-ANT1 (1:1000, 69569; Cell Signaling Technology, Danvers, USA), anti-Beclin-1 (1:1000, PB9076; Boster), anti-autophagy related protein (ATG)3 (1:1000, BM5103; Boster), anti-ATG5 (1:1000, BA3525-2; Boster), anti-ATG7 (1:1000, A00346; Boster), anti-ATG16L (1:1000, ATG16L; Boster), anti-LC3 (1:1000, PA01524; Boster), anti-P62 (1:2000, PB0458; Boster), anti-Bcl-2 (1:1000, BM0200; Boster), anti-BAX (1:5000, BA0315-2; Boster), anti-BAD (1:5000, BM4241; Boster), anti-PINK1 (1:1000, A00201-2; Boster), anti-Parkin (1:1000, BM4909; Boster), and anti-GAPDH (1:10,000, BM1623; Boster).

### Quantitative real-time polymerase chain reaction (qPCR)

Total RNA was extracted using Trizol reagent (15596-018; Invitrogen, Carlsbad, USA). The RNA was reverse transcribed into complementary deoxyribonucleic acid (cDNA) using the TransScript All-in-One First-Strand cDNA Synthesis SuperMix for qPCR kit (AT341; Transgen, Beijing, China). The amplification program included initial denaturation at 95°C for 3 min, followed by 45 cycles of denaturation at 95°C for 7 s, annealing at 57°C for 10 s, and extension at 72°C for 15 s. The relative expression of the target gene was normalized to the level of *GAPDH*, and the relative expression levels were calculated using the 2 ^–ΔΔCt^ method. *GAPDH* served as an internal control, and the experiment was repeated three times. The sequences of primers used were as follows: *ANT1*-forward: 5′-GGAGCGAGATCCCTCCAAAAT-3′, reverse: 5′-GGCTGTTGTCATACTTCTCATGG-3′; *PINK1*-forward: 5′-GGAGGAGTATCTGATAGGGCAG-3′, reverse: 5′-AACCCGGTGCTCTTTGTCAC-3′; and *GAPDH*-forward: 5′-CAAGGGGATGCTGCCTGACC-3′, reverse: 5′-TGTCCCCGTGTACATAATAT-3′.

### Cell proliferation assay

Cells in the logarithmic growth phase were seeded in a 96-well plate at a density of 5 × 10 ^3^ cells per well. The culture medium in the 96-well plate was replaced, and 10 μL of CCK-8 reagent (YEASEN, Shanghai, China) was added to each well. The plate was then incubated at 37°C in a cell culture incubator protected from light for 1 h. The absorbance at 450 nm was measured using a microplate reader (Biotek, Winooski, USA). The process was repeated at four time points—12, 24, 48, and 72 h after transfection—and the data were collected. A cell growth curve was plotted using the time points as the x-axis and the OD _450_ as the y-axis. The experiment was repeated three times, with three replicates for each group of cells.

### Colony formation assay

The cells in the logarithmic growth phase were trypsinized, resuspended in complete culture mediums, and counted. The cells were then seeded in a 6-well plate and cultured, with the medium changed every 3 days to maintain optimal growth conditions. The cell status was observed throughout the culture period. After 14 days, the cells were fixed with 4% paraformaldehyde for 30 min. Following fixation, the cells were stained with 0.5% crystal violet for 15 min. Colonies were photographed, and the number of colonies was quantified via ImageJ software (National Institutes of Health, Bethesda, USA).

### Transwell migration and invasion assays

Transwell chambers with 8.0 μm pore size membranes (Corning, New York, USA) were used according to the manufacturer’s protocol. The cells (1 × 10 ^5^ cells per well) were seeded in the upper chamber in 100 μL of serum-free medium, while 600 μL of complete culture medium was added to the lower chamber as a chemoattractant. After incubation at 37°C for 24 h, the residual cells on the upper surface of the membrane were removed with a cotton swab, and the cells on the lower surface of the membrane were considered migrated cells. For the transwell invasion assay, the procedure was similar to that for the migration assay described above, except that before the cells were seeded onto the membrane, 100 μL of Matrigel (BD, Franklin Lakes, USA) diluted 1:8 in DMEM was added to each well. The Matrigel was then incubated for 6 h at 37°C. The cells were subsequently cultured for an additional 48 h. The cells were fixed with 4% paraformaldehyde and stained with 0.1% crystal violet solution. Images of the cells that migrated through the filter were captured via an inverted light microscope (Olympus, Tokyo, Japan).

### Wound healing assay

A horizontal line was drawn on the back of a 6-well plate using a marker pen. Cells in the logarithmic growth phase were seeded into the wells at a density of 5 × 10 ^5^ cells per well and cultured in complete culture medium. Once the cells reached 100% confluence, a sterile pipette tip was used to create a scratch on the cell layer. The detached cells were washed away with PBS, and the cells were further cultured in serum-free medium for 48 h. The healing of the scratch was observed and photographed at 0 and 48 h post-scratching. The wound healing rate (%) was calculated as: wound healing rate (%) = [(wound width at 0 h – wound width at 48 h) / wound width at 0 h] × 100%. The experiment was repeated three times, and the results were averaged.

### Mitochondrial morphology analysis

The cells were seeded on coverslips in 6-well plates at a density of 1 × 10 ^5^ cells/well. After 24 h of culture, the cells were subjected to the corresponding treatments according to the experimental groups and then incubated further. Forty-eight hours later, pre-warmed MitoTracker Green staining solution (Beyotime, Shanghai, China) was added, and the cells were incubated at 37°C for 30 min. After incubation, the cells were fixed with 4% paraformaldehyde for 10 min. The cells were then washed three times with PBS, each lasting 3 min. Permeabilization solution was added, and the cells were incubated in the dark for 15 min. After another three washes with PBS (3 min each), DAPI staining was performed for 4 min, followed by three more PBS washes (3 min each). The coverslips were then mounted with fluorescence anti-fade reagent. Mitochondrial staining and cellular morphology were observed, and images were captured using a laser confocal microscope (Zeiss, Oberkochen, Germany).

### Measurement of cellular ROS levels

Cellular ROS production was measured via the oxidation-sensitive fluorescent probe DCFH-DA (Beyotime). Briefly, after the designated treatment, the cells were washed three times with PBS. Subsequently, 10 μM DCFH-DA was added to the cells, which were subsequently incubated at 37°C in a cell culture incubator for 20 min. The residual DCFH-DA in the cells was then washed away with PBS three times, and the cells were observed under an inverted fluorescence microscope (Carl Zeiss Jena GmbH, Jena, Germany) with an excitation wavelength of 488 nm and an emission wavelength of 530 nm. The intensity of green fluorescence served as an indicator of the ROS level and was analyzed via ImageJ software. Additionally, relative ROS levels were detected via flow cytometry after the cells were collected.

### Measurement of the mitochondrial membrane potential (Δψm)

The measurement of the mitochondrial membrane potential was performed via an enhanced mitochondrial membrane potential assay kit with JC-1 (C2003S; Beyotime). After being washed with PBS, the cells were incubated with JC-1 solution at 37°C for 30 min. After the samples were washed twice with PBS, the changes in the mitochondrial membrane potential (ratio of red to green fluorescence) were analyzed by flow cytometry.

### Flow cytometry

The cells were seeded in a 6-well plate, and when the cell confluence reached 50% to 70%, the cells were subjected to the experimental treatments. After 48 h, the cells from each group were collected via centrifugation, ensuring a cell count of 5 × 10 ^5^ cells per group. The cells were resuspended in 500 μL of 1× binding buffer. Then, 5 μL of Annexin V-FITC was added to each tube, followed by gentle mixing, and 10 μL of PI was added. After slow vortexing, the cells were incubated at room temperature in the dark for 5 min. Annexin V-FITC (Ex = 488 nm; Em = 530 nm) was detected in the FITC channel (usually FL1), and PI was detected in the PE channel (usually FL2) using a flow cytometer.

### LC3 immunofluorescence

For LC3 immunofluorescence analysis, the cells were fixed with 4% paraformaldehyde and permeabilized with 0.1% Triton X-100. LC3B was stained with 0.3 μg/mL anti-LC3B antibody (autophagosome marker; ab48394; Abcam, Cambridge, UK). The primary antibody was incubated at room temperature for 3 h, followed by incubation with NorthernLight™ 557-conjugated anti-rabbit IgG secondary antibody (NL004; R&D Systems, Minneapolis, USA). DAPI (blue) counterstaining was performed to visualize the cell nuclei. The cell nuclei appeared as blue fluorescence, while LC3 appeared as red fluorescence.

### Acridine orange staining

The cells were seeded on coverslips in 6-well plates at a density of 1 × 10 ^5^ cells/well. After 24 h of culture, the cells were treated according to the experimental groups and incubated further. After 48 hours, the cells were digested with trypsin and washed twice with PBS. The cells were then incubated in PBS containing 1 μg/mL acridine orange staining solution for 15 min in the incubator. After incubation, the cells were washed three times with PBS. The fluorescence intensity was then analyzed by flow cytometry.

### Animal experiments

SW620 cells transfected with shANT1#1 were used to generate a xenograft model. Twenty-four female BALB/c-nu mice, aged 4–6 weeks and weighing 18–20 g, were procured from Sipeifu Biotech (Beijing, China). For the subcutaneous tumor formation assay, 4 × 10 ^6^ cells in 100 μL of PBS were injected subcutaneously into nude mice (8 mice per group). The tumor volume was measured weekly. After 4 weeks, all the mice were euthanized via intraperitoneal injection of pentobarbital sodium (100 mg/kg), and the tumors were dissected. All animal experiment procedures were conducted in accordance with the Guidelines for the Care and Use of Laboratory Animals and were approved by the Yangzhou University Laboratory Animal Ethics Committee (approval No. 202307022). Animal interventions were not blinded, but sample analysis was conducted blindly.

### Hematoxylin and eosin (HE) staining

Tumor tissues from nude mice were fixed in 10% formaldehyde, trimmed, routinely embedded in paraffin, and sectioned into 4-μm-thick slices. The sections were deparaffinized, rehydrated, and stained with hematoxylin for 4–8 min, followed by rinsing in running water. The sections were differentiated in a 1% hydrochloric acid/ethanol solution, thoroughly washed, and then stained with eosin for 2–3 min. After dehydration and clearing, the slides were mounted and air-dried. Pathological changes in the tissues were observed under a light microscope, and images were captured.

### Ki67 immunohistochemistry

Paraffin sections were deparaffinized and rehydrated as usual, followed by immersion in 1× sodium citrate solution for antigen retrieval using microwave heating. The sections were incubated in 3% hydrogen peroxide to quench endogenous peroxidase activity and then blocked with 5% BSA at 37°C for 30 min. An anti-Ki67 primary antibody (1:200; Cell Signaling Technology) was applied, and the sections were incubated overnight at 4°C. After three washes with PBS (5 min each), the sections were incubated with a HRP-conjugated secondary antibody (Abcam, Cambridge, UK) at 37°C for 30 min, followed by washing with PBS. DAB (KeyGEN BioTECH, Nanjing, China) was used for color development, and the sections were counterstained with hematoxylin for 10 s and then rinsed with tap water. Positive cells were observed under a light microscope.

### TUNEL staining

Paraffin sections of tumor tissues were deparaffinized and rehydrated. Proteinase K (20 μg/mL) was added, and the sections were incubated at 37°C for 30 min, followed by incubation at room temperature for 20 min. After washing with PBS, 50 μL of TUNEL detection solution (Servicebio, Wuhan, China) was added, and the sections were incubated at room temperature for 1 h. A peroxidase converter was then added, and the sections were incubated for an additional 30 min, followed by washing with PBS. DAB was used for color development, and the sections were counterstained with hematoxylin. After dehydration, clearing, and mounting with neutral balsam, the sections were observed and photographed under an inverted fluorescence microscope (Olympus).

### Statistical analysis

Statistical analysis was performed using SPSS 22.0 software. The measurement data are presented as the mean ± standard deviation. One-way analysis of variance was used to test for differences in means across multiple groups. The relationships between ANT1 expression and the survival/recurrence outcomes of patients were analyzed by plotting Kaplan-Meier survival curves. All tests were two-sided, and a significance level was considered statistically significant if *P* is less than 0.05.

## Results

### High expression of ANT1 in CRC is associated with poor prognosis

**Figure 1:**
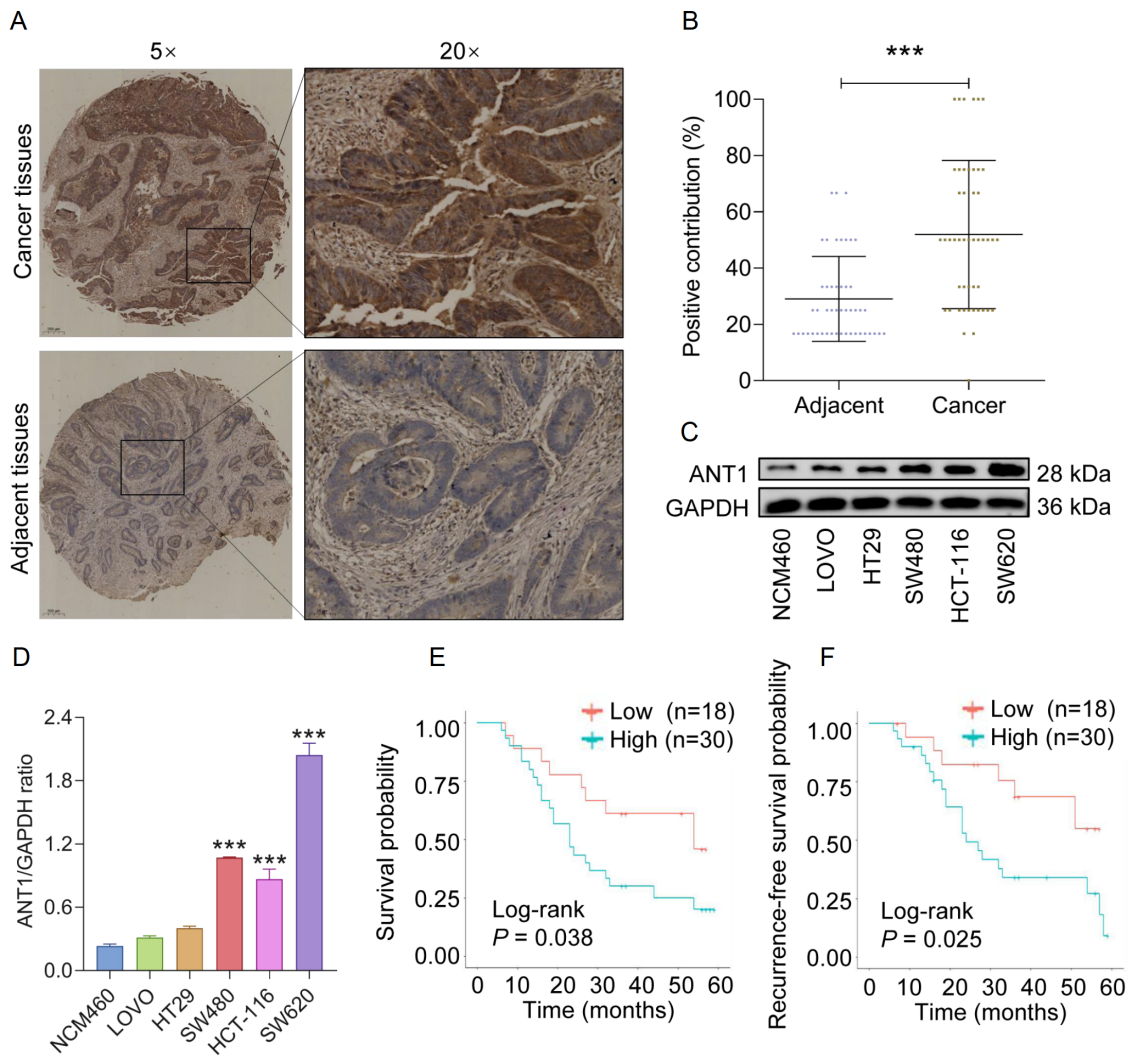
Figure 1 Upregulation of ANT1 in colorectal cancer and its association with poor prognosis (A,B) Representative images of ANT1 expression (A) and quantitative analysis of IHC staining (B). (C,D) Western blot analysis of ANT1 expression in different cell lines (n = 3). (E) Patients with low expression of ANT1 have significantly better overall survival than patients with high expression of ANT1. (F) Patients with high expression of ANT1 have significantly higher postoperative recurrence rates than patients with low expression of ANT1.

To investigate the potential role of ANT1 in CRC, we performed IHC analysis on tissue microarrays, including cancer tissues and matched adjacent normal tissues from 48 patients. The results revealed that positive staining for the ANT1 protein was located mainly in the cytoplasm of the tumor cells. Compared with those in the corresponding adjacent normal tissues, the expression levels of ANT1 were significantly greater in the CRC tissues ( Table 2 and Figure 1A,B). Additionally, the protein levels of ANT1 in cultured CRC cell lines (LoVo, HT-29, HCT-116, SW480, and SW620) were significantly higher than those in the human normal colon epithelial cell line NCM460 ( Figure 1C,D). The clinicopathological features of these patients are summarized in Table 3. The results obtained from the Kaplan-Meier survival curve revealed that the median survival period for patients in the high ANT1 expression group was 24 months, whereas it was 54 months for patients in the low ANT1 expression group ( *P*  < 0.05; Figure 1E). The median recurrence-free survival periods for patients with high and low ANT1 expression were 24 months and over 60 months, respectively ( *P*  < 0.05; Figure 1F). These findings collectively indicate the upregulation of ANT1 expression in CRC, which is associated with an unfavorable prognosis.

**Table:** **Table 2** ANT1 expression in colon cancer tissues and the adjacent tissues

	ANT1 expression		χ ^2^ value	*P* value
	Low (%)	High (%)			
Colon cancer	37.5	62.5		17.043	< 0.001
Adjacent tissues	79.2	20.8

**Table:** **Table 3** Association between clinicopathological characteristics and positive expression of ANT1 in colon cancer patients

Clinical parameter	Total [cases (%)]	ANT1 expression level [cases (%)]		χ ^2^	*P*
	Low	High			
Total	48	18 (37.5)	30 (62.5)			
Age (Years)					0.071	0.790
< 60	11	5 (45.5)	6 (54.5)			
≥ 60	37	13 (35.1)	24 (64.9)			
Gender					0.091	0.762
Female	20	8 (40.0)	12 (60.0)			
Male	28	10 (35.7)	18 (64.3)			
Tumor location					0.566	0.452
Rectum	23	6 (26.1)	17 (73.9)			
Left-sided colon	4	3 (75.0)	1 (25.0)			
Right-sided colon	9	5 (55.6)	4 (44.4)			
Sigmoid colon.	12	4 (33.3)	8 (66.7)			
Tumor size (cm)					1.318	0.251
< 4	14	7 (50.0)	7 (50.0)			
≥ 4	34	11 (32.4)	23 (67.6)			
Morphological subtypes					1.150	0.284
Ulcerative type	31	10 (32.3)	21 (67.7)			
infiltrative type	5	2 (40.0)	3 (60.0)			
protruding type	12	6 (50.0)	6 (50.0)			
Neural invasion					1.440	0.230
Negative	40	13	27			
Positive	8	5	3			
Vascular invasion					0.000	1.000
Negative	36	13	23			
Positive	12	5	7			
T stage					1.440	0.230
T1–T2	8	1 (12.5)	7 (87.5)			
T3–T4	40	17 (42.5)	23 (57.5)			
N stage					1.651	0.199
N0–N1	38	12 (31.6)	26 (68.4)			
N2–N3	10	6 (60.0)	4 (40.0)			
M stage					1.667	0.197
M0	47	17 (36.2)	30 (63.8)			
M1	1	1 (100.0)	0 (0.0)			
TNM stage					3.155	0.076
I	7	1 (14.3)	6 (85.7)			
II	25	9 (36.0)	16 (64.0)			
III	15	7 (46.7)	8 (53.3)			
IV	1	1 (100.0)	0 (0.0)

### Knockdown of *ANT1* inhibits CRC cell proliferation, invasion, and migration

**Figure 2:**
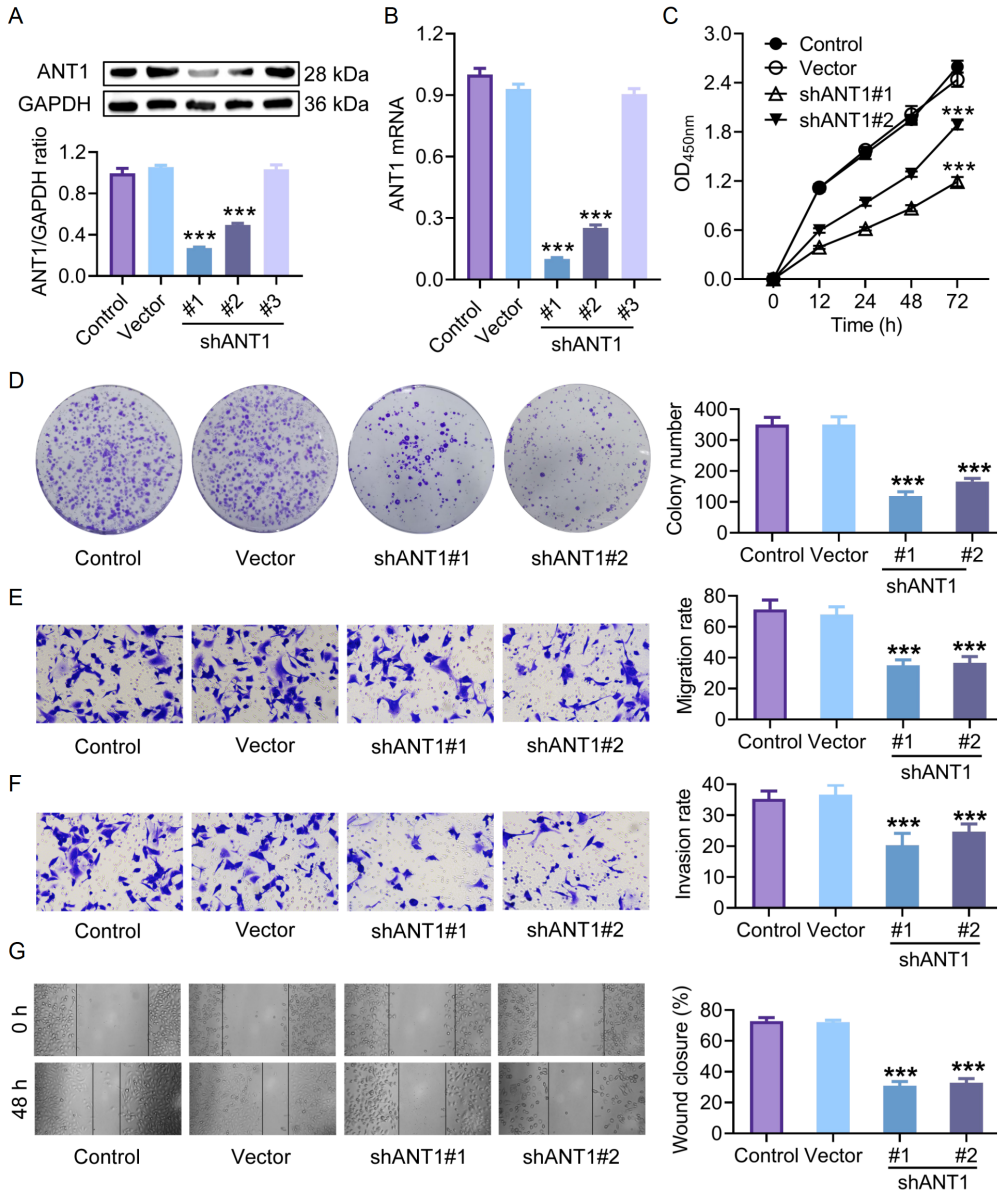
Figure 2 Knockdown of *ANT1* inhibits the proliferation, invasion, and migration of SW620 cells (A,B) Western blot analysis (A) and qRT-PCR (B) results demonstrated effective inhibition of ANT1 expression by shANT1#1 and shANT1#2 (n = 3). (C) Cell viability results from the CCK-8 assay revealed the reduced cell viability in the clones with ANT1 knockdown by shANT1#1 and shANT1#2 (n = 3). (D) The results from the adherent-independent colony formation assay revealed fewer colonies formed by SW620 cells transfected with shANT1#1 or shANT1#2 than by those in the control group (n = 3). (E,F) Both the shANT1#1 and shANT1#2 groups exhibited slower cell (E) migration and (F) invasion than did the control group (n = 3). (G) Wound healing assays demonstrated that shANT1#1 and shANT1#2 reduced the migration ability of SW620 cells (n = 3). Compared with the control group, ***P < 0.001.

**Figure 3:**
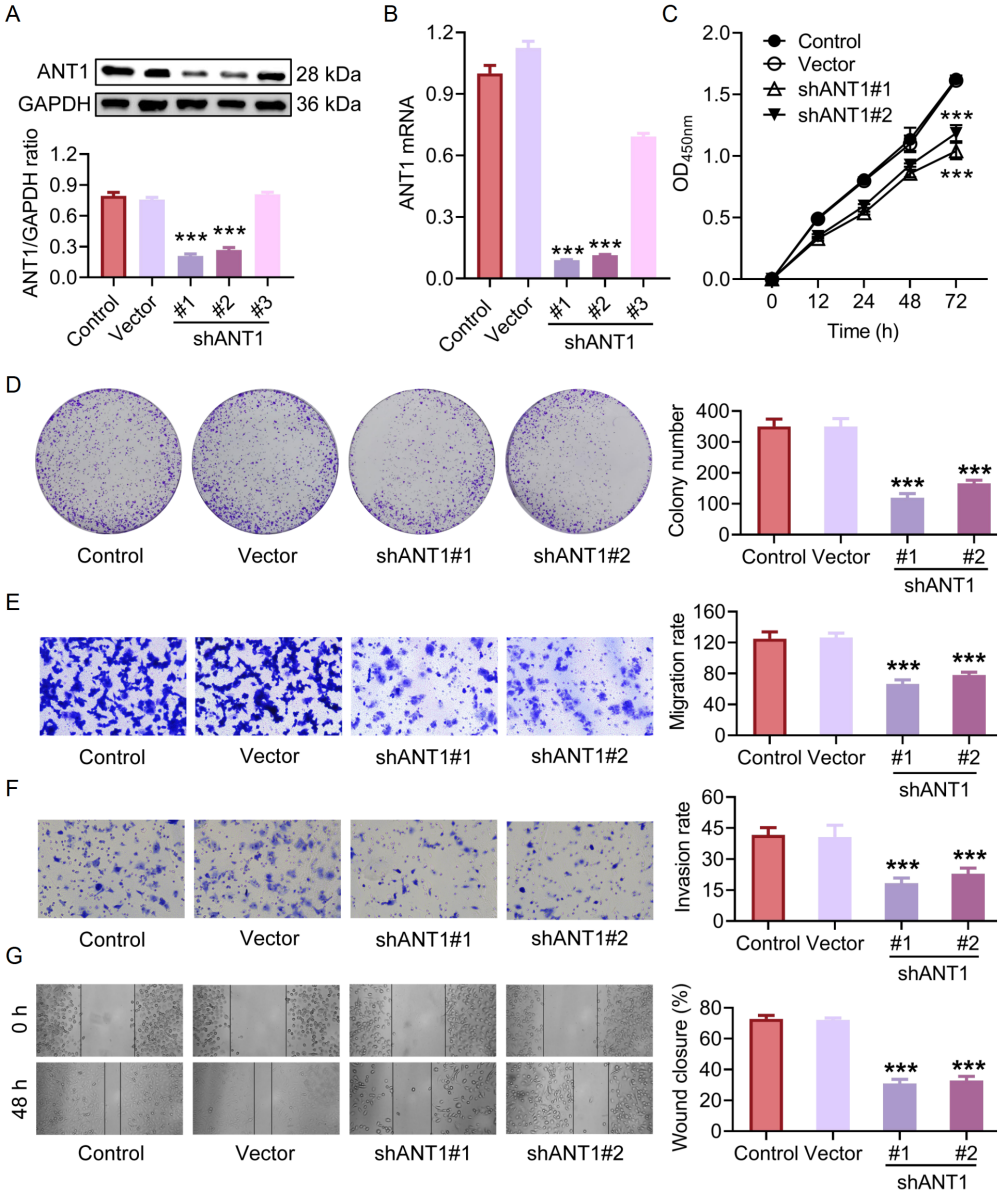
Figure 3 Knockdown of *ANT1* inhibits the proliferation, invasion, and migration of SW480 cells (A,B) Western blot analysis (A) and qRT-PCR (B) results demonstrated effective inhibition of ANT1 expression by shANT1#1 and shANT1#2 (n = 3). (C) Cell viability results from the CCK-8 assay revealed reduced cell viability in the clones with ANT1 knockdown by shANT1#1 and shANT1#2 (n = 3). (D) The results from the adherent-independent colony formation assay revealed fewer colonies formed by SW480 cells transfected with shANT1#1 or shANT1#2 than by those in the control group (n = 3). (E,F) Both the shANT1#1 and shANT1#2 groups exhibited slower cell (E) migration and (F) invasion than did the control group (n = 3). (G) Wound healing assays demonstrated that shANT1#1 and shANT1#2 reduced the migration ability of SW480 cells (n = 3). Compared with the control group, ***P < 0.001.

To evaluate the functional role of ANT1 in CRC, we transfected three different vector-based ANT1-specific short hairpin RNAs (shANT1#1, #2, and #3) into SW620 cells and selected stable cell clones in which *ANT1* was knocked out using puromycin. Western blot analysis revealed that, compared with the empty vector, shANT1#1 and shANT1#2 successfully reduced the expression levels of ANT1 in SW620 cells ( Figure 2A,B). We subsequently assessed the proliferation, migration, and invasion capacity of SW620 cells with *ANT1* knockdown via shANT1#1 and shANT1#2. The OD _450nm_ value is directly proportional to cell viability. The absorbance data from the CCK-8 assay demonstrated that the viability of SW620 cells transfected with shANT1#1 or shANT1#2 was significantly lower than that of the vector group ( *P*  < 0.05; Figure 2C). Colony formation assay revealed that the number of colonies formed by SW620 cells transfected with shANT1#1 or shANT1#2 was lower than that formed by control cells ( *P*  < 0.05; Figure 2D). Both the shANT1#1 and shANT1#2 groups exhibited slower cell migration and invasion than did the control group ( Figure 2E,F). The wound healing assay also revealed that *ANT1* knockdown decreased the migration ability of SW620 cells ( Figure 2G). Similar results were obtained with SW480 cancer cells ( Figure 3). These data suggest that *ANT1* knockdown inhibits the proliferation, invasion, and migration of CRC cells.

### Knockdown of *ANT1* enhances the accumulation of damaged mitochondria, increases ROS production, and induces apoptosis in CRC cells

**Figure 4:**
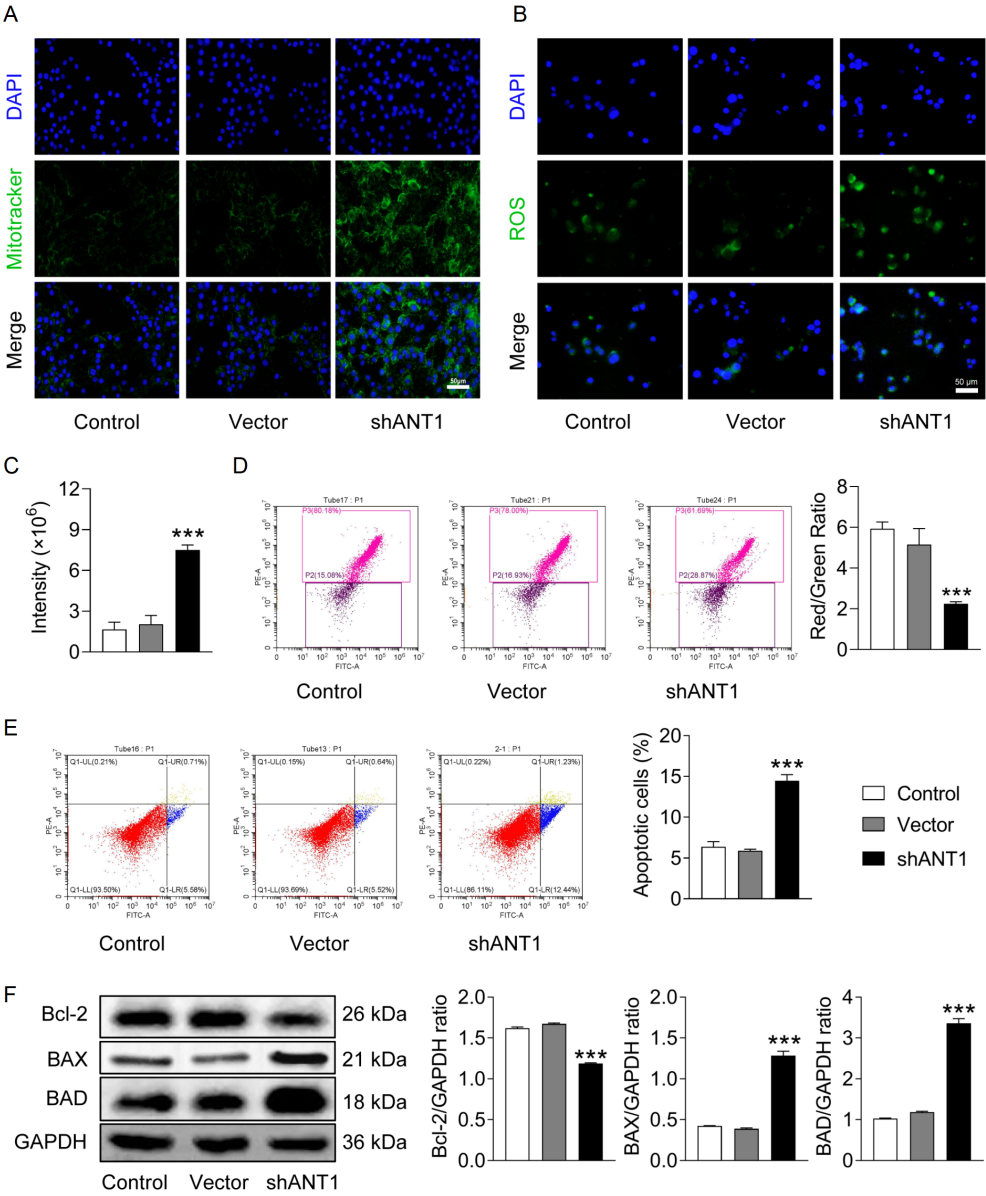
Figure 4 Knockdown of *ANT1* enhances the accumulation of damaged mitochondria, increases ROS production, and induces apoptosis in CRC cells (A) MitoTracker staining showing mitochondrial accumulation (n = 3). (B) Immunofluorescence staining was performed to measure the ROS levels (magnification: ×200). (C) Positive rate of ROS staining (n = 3). (D) Flow cytometry analysis was used to assess the mitochondrial membrane potential (n = 3). (E) Flow cytometry analysis was used to determine the percentage of apoptotic cells (n = 3). (F) Western blot analysis was performed to assess the expression levels of apoptosis-related proteins (n = 3). Compared with the control group, ***P < 0.001.

We subsequently transfected shANT1#1, which exhibited the most efficient knockdown, into SW620 cells to further investigate the direct effect of ANT1 on mitochondrial function. As shown in Figure 4A, MitoTracker staining revealed a significant increase in green fluorescence intensity following shANT1 transfection, indicating the accumulation of damaged mitochondria that were not cleared. It is well known that mitochondria are the main site of ROS production in cells. The DCFH-DA assay revealed a significant increase in green fluorescence intensity in shANT1-transfected SW620 cells compared with control cells ( *P*  < 0.001), indicating that *ANT1* knockdown induced ROS production in SW620 cells ( Figure 4B,C). In addition, the red/green ratio was significantly lower in the shANT1 group than in the control group, suggesting that the mitochondrial membrane potential was decreased, resulting in the inability of JC-1 to exist in the mitochondrial matrix as a polymer ( Figure 4D). Altered mitochondrial function plays a crucial role in the development of apoptosis. Flow cytometry analysis revealed a significant increase in the proportion of apoptotic shANT1-transfected SW620 cells, indicating that *ANT1* knockdown could induce apoptosis in SW620 cells ( Figure 4E). The western blot analysis results revealed that *ANT1* knockdown downregulated the expression of the anti-apoptotic protein Bcl-2 and upregulated the expression of the pro-apoptotic proteins BAX and BAD, which led to the induction of apoptosis ( Figure 4F). These findings suggest that silencing of *ANT1* may impair mitochondrial function, increase ROS production, and ultimately induce apoptosis. Together with the accumulation of mitochondria, these results suggest that mitophagy may be inhibited in the shANT1 group, exacerbating mitochondrial dysfunction.

### Knockdown of *ANT1* suppresses mitophagy in CRC cells

**Figure 5:**
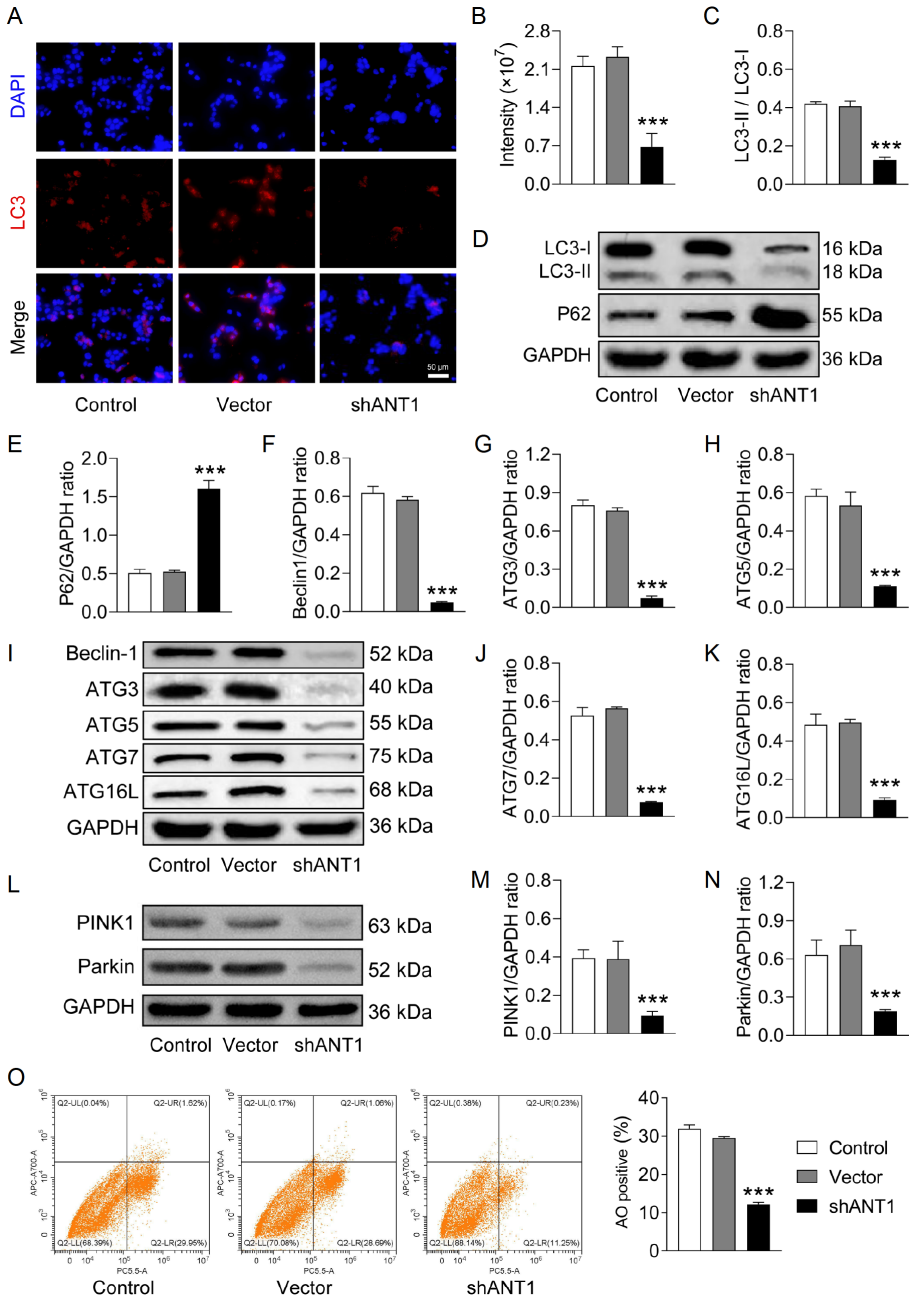
Figure 5 Knockdown of *ANT1* suppresses mitophagy in CRC cells (A) Immunofluorescence staining was performed to detect changes in LC3 (magnification: ×200). (B) Positive rate of LC3 staining (n = 3). (C–E) Western blot analysis was conducted to examine the expression levels of autophagy markers (n = 3). (F–K) Western blot analysis of the expression levels of Beclin1, ATG3, ATG5, ATG7, and ATG16L (n = 3). (L–N) Western blot analysis of PINK1 and Parkin protein expression levels (n = 3). (O) Acridine orange flow cytometry showing the number of acidic autophagic vesicles (n = 3). Compared with the control group, ***P < 0.001.

To further investigate the role of mitophagy in *ANT1* knockdown, various markers were used to analyze the process in detail. The immunofluorescence results revealed varying degrees of bright, punctate red fluorescence in the control and vector groups, indicating the transformation of LC3-I to LC3-II and the activation of autophagy. In contrast, the shANT1 group exhibited only minimal red fluorescence, suggesting the inhibition of autophagy ( Figure 5A,B). Similarly, the ratio of LC3-II/LC3-I was decreased, and the protein expression of P62 was increased in shANT1-transfected SW620 cells compared with those in the control group ( Figure 5C–E). Additionally, the expressions of Beclin1, ATG3, ATG5, ATG7, and ATG16L, crucial autophagy proteins, were significantly reduced, indicating an impaired autophagy pathway and weakened autophagic activity ( Figure 5F–K). The expression levels of the mitochondrial autophagy-related proteins PINK1 and Parkin were also notably downregulated in the shANT1 group ( Figure 5L–N), suggesting that the PINK1/Parkin pathway was dysfunctional and that mitochondrial autophagy was not effectively executed. Acridine orange flow cytometry revealed a reduced number of acidic autophagic vesicles in the shANT1 group ( Figure 5O), further confirming the decline in autophagic activity. Collectively, these findings indicate that silencing of *ANT1*significantly inhibits the progression of mitochondrial autophagy.

### Overexpression of PINK1 rescues the impact of *ANT1* knockdown on CRC cell proliferation, invasion, and migration

**Figure 6:**
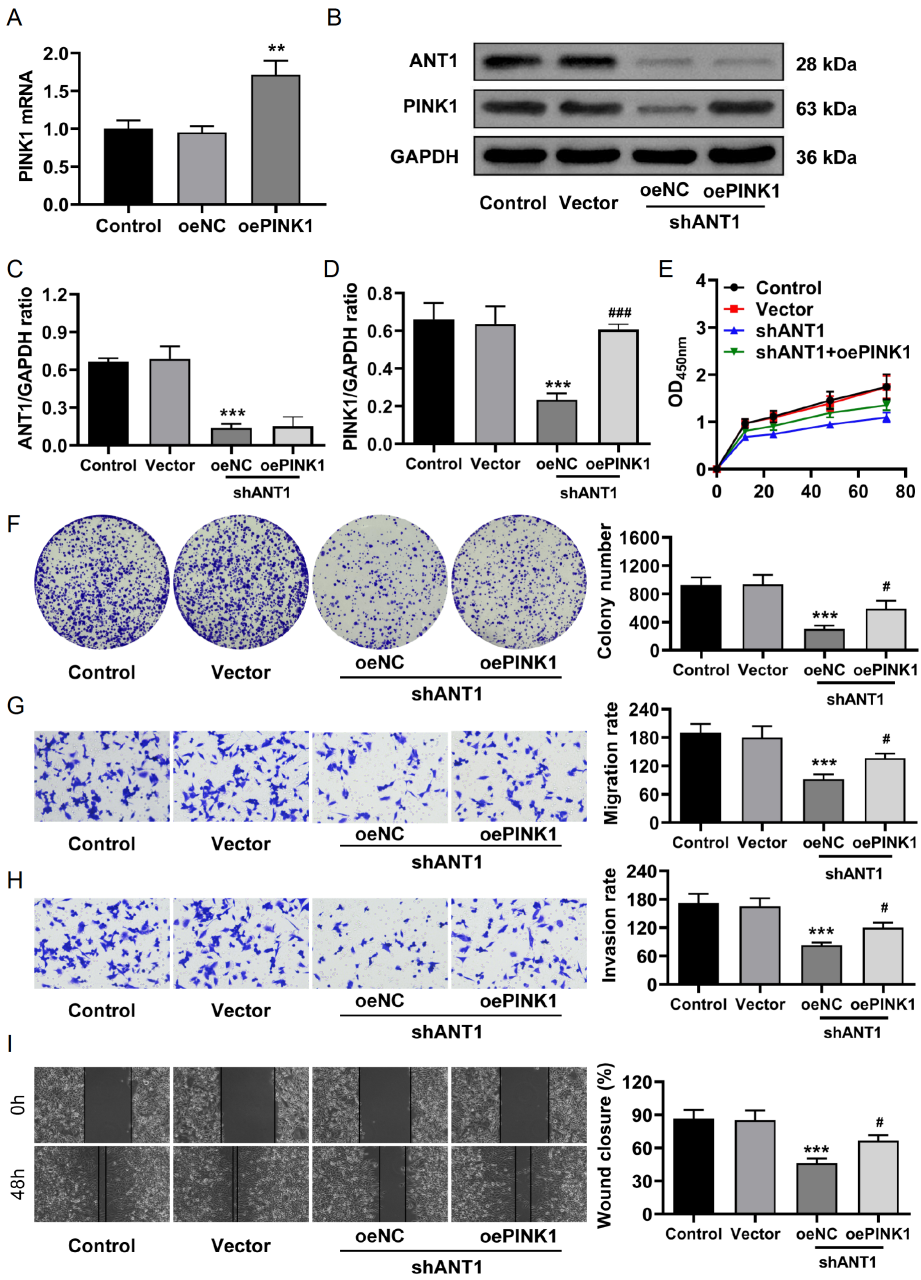
Figure 6 Overexpression of *PINK1* rescues the impact of *ANT1* knockdown on CRC cell proliferation, invasion, and migration (A) qRT-PCR validation of the effectiveness of PINK1 overexpression. (B–D) Western blot analysis of ANT1 and PINK1 protein expression levels (n = 3). (E) Cell viability was assessed by a CCK-8 assay (n = 3). (F) A nonadherent colony formation assay was used to evaluate cell proliferation ability (n = 3). (G,H) Transwell migratio (G) and invasion (H) assays were performed to assess cell migration and invasion abilities. (I) Wound healing assay showing the migration abilities of different groups of cells (n = 3). Compared with the control group, ***P < 0.001; compared with the shANT1 + oeNC group, # P < 0.05, ### P < 0.001.

To further investigate the role of the mitochondrial autophagy protein PINK1 in the anti-CRC effects of *ANT1* knockdown, we overexpressed PINK1 in *ANT1*-knockdown cells. First, we validated the effectiveness of PINK1 overexpression (oePINK1) through PCR, which revealed significant upregulation of *PINK1* mRNA levels in the oePINK1 group ( Figure 6A), indicating successful construction of the overexpression system. In the rescue experiments, PINK1 overexpression rescued the downregulation of PINK1 caused by *ANT1* knockdown without affecting ANT1 expression levels ( Figure 6B–D). Next, a series of functional assays were conducted to evaluate the impact of PINK1 overexpression on the proliferation, invasion, and migration of shANT1 cells. The results of the CCK-8 and colony formation assays demonstrated that PINK1 overexpression significantly restored the inhibition of cell proliferation observed in the shANT1 group ( Figure 6E,F). Transwell migration and invasion assays revealed that the migration and invasion capacities of cells in the shANT1 group were impaired; however, PINK1 overexpression partially restored these abilities ( Figure 6G,H). Additionally, wound healing assays revealed that PINK1 overexpression significantly promoted wound healing in shANT1 cells, which exhibited reduced migration ( Figure 6I). These results collectively suggest that PINK1 overexpression can mitigate the negative effects of *ANT1* knockdown on CRC cell proliferation, invasion, and migration.

### PINK1 overexpression alleviates the impact of *ANT1* knockdown on mitochondrial function, ROS production, and apoptosis

**Figure 7:**
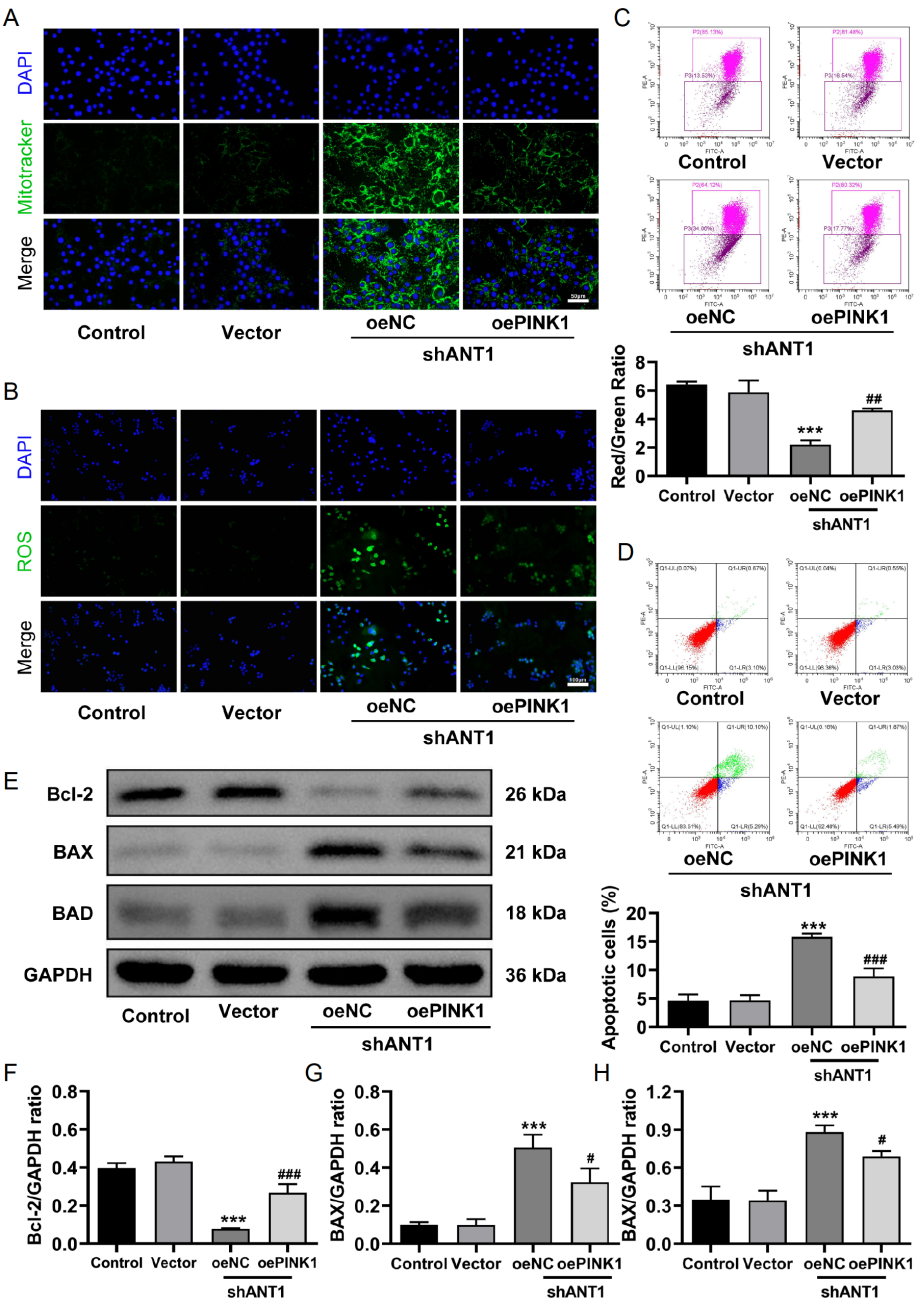
Figure 7 PINK1 overexpression alleviates the impact of *ANT1* knockdown on mitochondrial function, ROS production, and apoptosis (A) MitoTracker fluorescence staining showing mitochondrial accumulation (n = 3). (B) DCFH-DA fluorescence was used to detect ROS production (n = 3). (C) JC-1 staining showing changes in the mitochondrial membrane potential (n = 3). (D) Flow cytometry was used to detect apoptosis (n = 3). (E–H) Western blot analysis of the expressions of apoptosis-related proteins (n = 3). Compared with the control group, ***P < 0.001; compared with the shANT1 + oeNC group, # P < 0.05, ## P < 0.01, ### P < 0.001.

The results of MitoTracker staining revealed a decrease in green fluorescence intensity in the shANT1 + oePINK1 group, indicating that PINK1 overexpression can reduce abnormal mitochondrial accumulation ( Figure 7A). JC-1 staining further confirmed the recovery of the mitochondrial membrane potential. Compared with that in the shANT1 + oeNC group, the red/green ratio in the shANT1 + oePINK1 group was significantly greater, indicating that PINK1 overexpression can improve the loss of the mitochondrial membrane potential ( Figure 7B). Additionally, DCFH-DA fluorescence staining revealed a significant reduction in ROS generation in the shANT1 + oePINK1 group ( Figure 7C), suggesting that PINK1 overexpression can alleviate the excessive ROS production caused by *ANT1* knockdown. Flow cytometric analysis of apoptosis revealed that the apoptosis rate in the shANT1 + oePINK1 group was significantly lower than that in the shANT1 + oeNC group, suggesting that PINK1 overexpression can mitigate the apoptosis induced by *ANT1* knockdown ( Figure 7D). Moreover, Western blot analysis revealed that the expression of the anti-apoptotic protein Bcl-2 was restored in the shANT1 + oePINK1 group, whereas the expression levels of the pro-apoptotic proteins BAX and BAD decreased ( Figure 7E–H), further demonstrating that PINK1 overexpression can rescue the apoptosis triggered by *ANT1* knockdown. These data collectively indicate that PINK1 overexpression can partially reverse the negative effects of *ANT1* knockdown on CRC cells by restoring mitochondrial function, reducing ROS production, and inhibiting apoptosis.

### Overexpression of PINK1 restores the inhibition of mitophagy induced by *ANT1* knockdown

**Figure 8:**
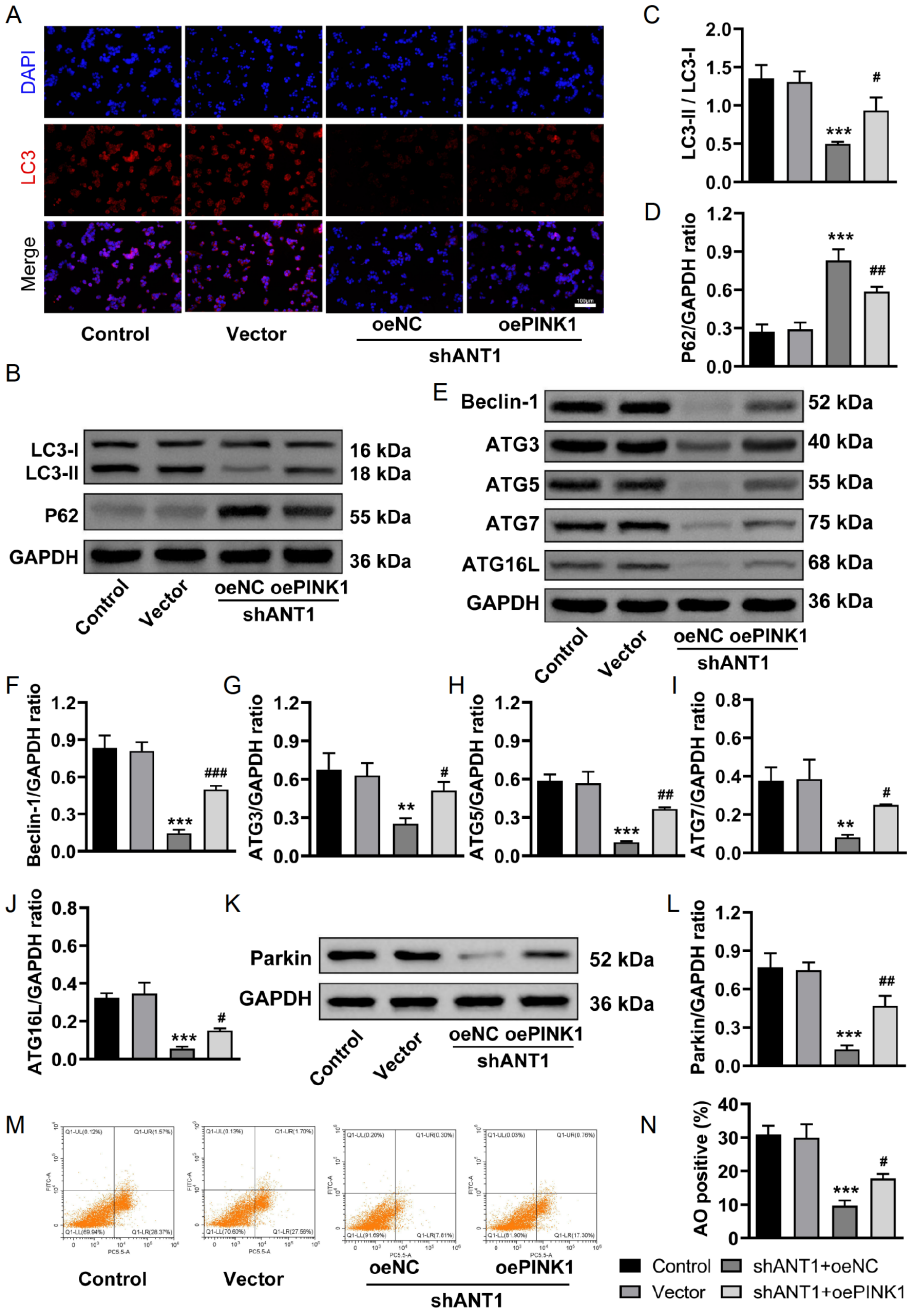
Figure 8 PINK1 overexpression restores the inhibition of mitophagy induced by *ANT1* knockdown (A) Immunofluorescence detection of LC3 expression (n = 3). (B–D) Western blot analysis of the LC3-II/LC3-I ratio and P62 protein expression (n = 3). (E–J) Western blot analysis of the expressions of the autophagy-related proteins Beclin1, ATG3, ATG5, ATG7, and ATG16L (n = 3). (K,L) Western blot analysis of Parkin expression (n=3). (M,N) Acridine orange flow cytometry was used to detect the number of acidic autophagic vesicles (n = 3). Compared with the control group, **P < 0.01, ***P < 0.001; compared with the shANT1 + oeNC group, # P < 0.05, ## P < 0.01, ### P < 0.001.

In the context of PINK1 overexpression, we investigated whether it could reverse the inhibition of mitochondrial autophagy caused by *ANT1* knockdown. Immunofluorescence experiments revealed a significant reduction in the red fluorescence of LC3 in the shANT1 group, indicating that autophagy was suppressed. In contrast, the shANT1 + oePINK1 group exhibited partial recovery of red fluorescence signals, suggesting that PINK1 overexpression can promote the conversion of LC3-I to LC3-II and restore autophagy activity ( Figure 8A). Western blot analysis revealed a significant decrease in the LC3-II/LC3-I ratio and an increase in P62 protein expression in the shANT1 group. However, in the shANT1 + oePINK1 group, the LC3-II/LC3-I ratio was restored, and P62 protein expression was significantly decreased ( Figure 8B–D), indicating that PINK1 overexpression positively regulates autophagy activity. Additionally, the expressions of the autophagy-related proteins Beclin1, ATG3, ATG5, ATG7, and ATG16L were significantly reduced in the shANT1 group, but their expressions were restored following PINK1 overexpression ( Figure 8E‒J). The expression level of Parkin, which is closely associated with mitochondrial autophagy, was also significantly inhibited by *ANT1* knockdown, but it markedly increased in the context of PINK1 overexpression, suggesting partial recovery of PINK1/Parkin pathway function ( Figure 8K,L). Acridine orange flow cytometry results similarly revealed a decrease in the number of acidic autophagic vesicles in the shANT1 group, whereas the shANT1 + oePINK1 group presented a significant increase in the number of these vesicles ( Figure 8M,N), further confirming that PINK1 overexpression can restore mitochondrial autophagy activity. These results suggest that PINK1 overexpression can partially reverse the inhibition of mitochondrial autophagy caused by *ANT1* knockdown by restoring the PINK1/Parkin pathway.

### Knockdown of *ANT1* inhibits tumor growth in SW620 xenograft mice via impairment of the mitophagy pathway

**Figure 9:**
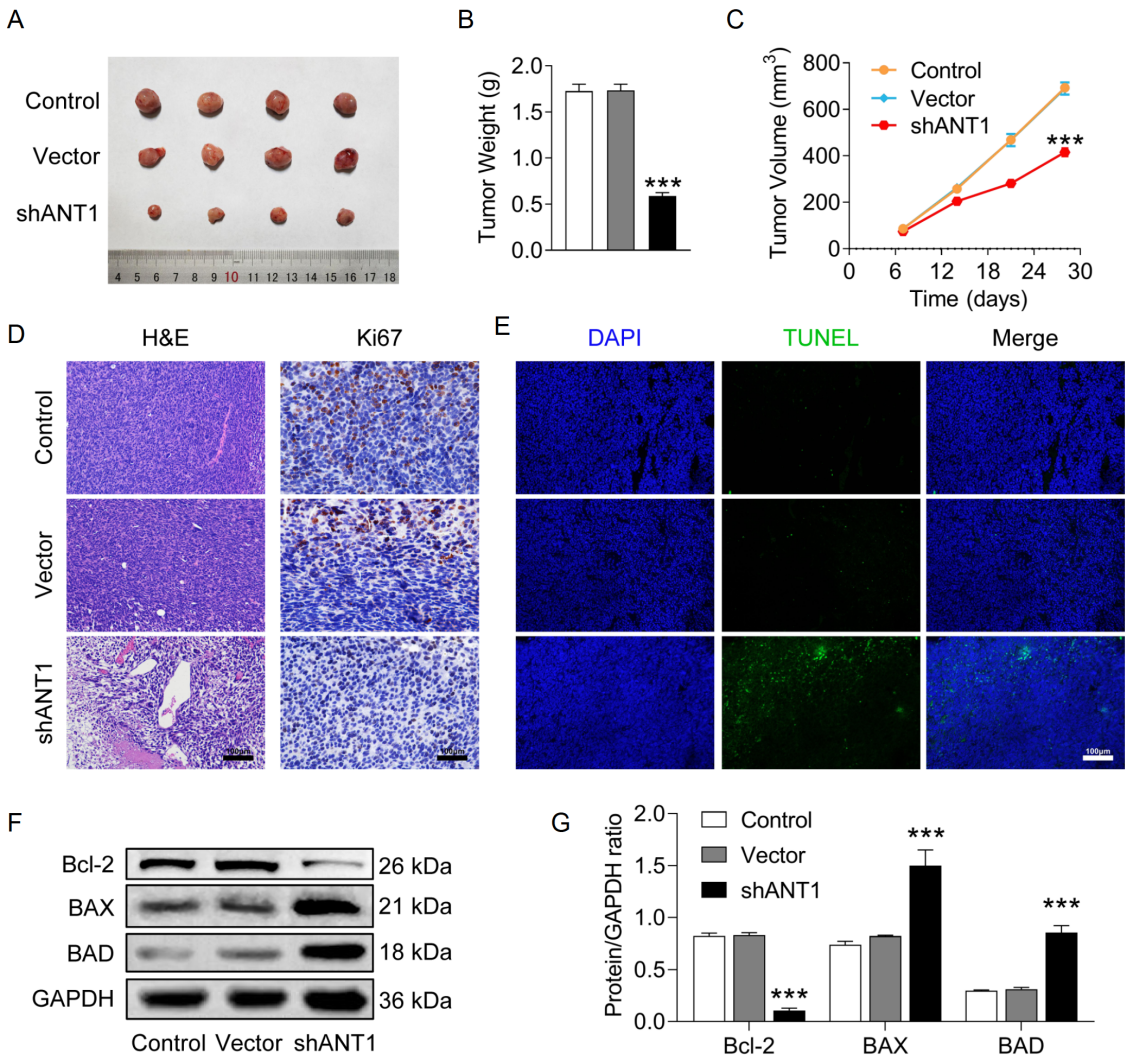
Figure 9 Knockdown of *ANT1* inhibits tumor growth in SW620 xenograft mice (A) Picture of xenograft tumors in each group. (B) Tumor weight. (C) Tumor volume curve. (D) Representative images of HE staining, Ki67 immunohistochemistry, and TUNEL fluorescence staining of tumor tissues (n = 3). (E,F) Western blot analysis was performed to assess the expression levels of apoptosis-related proteins (n = 3). Compared with the control group, ***P < 0.001.

**Figure 10:**
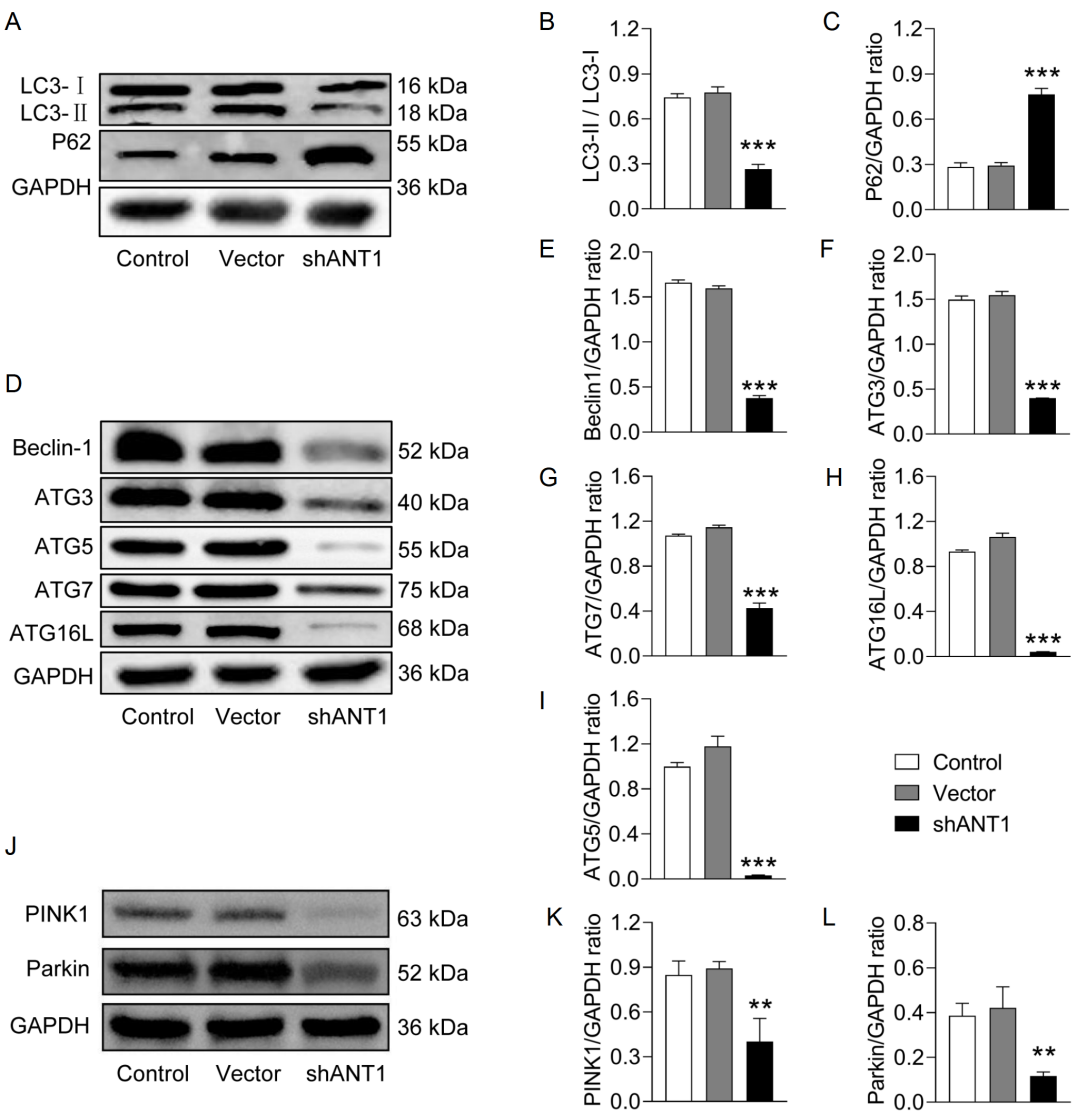
Figure 10 Knockdown of *ANT1* inhibits mitophagy in SW620 xenograft tumors (A–C) Western blot analysis of the LC3-II/LC3-I ratio and P62 protein expression levels (n = 3). (D–I) Western blot analysis of Beclin1, ATG3, ATG5, ATG7, and ATG16L expression levels (n = 3). (J–L) Western blot analysis of PINK1 and Parkin protein expression levels (n = 3). Compared with the control group, **P < 0.01, ***P < 0.001.

A SW620 xenograft mouse model was established to assess the effect of ANT1 on tumor growth. The results revealed that after 28 days, the tumor volume and weight were significantly lower in the shANT1 group than in the control group ( Figure 9A‒C). As shown in Figure 9D, the tumor tissue in the shANT1 group exhibited a markedly lower cell density than that in the control group did, suggesting inhibited growth of tumor cells. Ki67 immunohistochemistry revealed that the proliferation index in the shANT1 group was significantly lower than that in the control group, further confirming the suppression of cell proliferation. TUNEL fluorescence staining revealed a significant increase in the number of apoptotic cells in the shANT1 group, supporting the role of ANT1 in cancer cell survival ( Figure 9E). Moreover, *ANT1* knockdown also induced apoptosis in SW620 xenograft tumor cells. An examination of the expression levels of apoptosis-related proteins revealed the upregulation of BAX and BAD and the downregulation of Bcl-2 in the shANT1 group, indicating that ANT1 promoted apoptosis ( Figure 9F,G). *ANT1* knockdown significantly decreased the LC3-II/LC3-I ratio, indicating the inhibition of LC3-I conversion to LC3-II ( Figure 10A,B), thereby affecting normal autophagy. Additionally, the protein expression of P62, an autophagy-related protein, was significantly greater in the shANT1 group than in the control group ( Figure 10C). This increase may be attributed to impairment of the autophagy pathway, leading to the inability to degrade P62 properly. Further western blot analysis revealed the downregulation of autophagy-related proteins, such as Beclin-1, ATG3, ATG5, ATG7, and ATG16L, under conditions of *ANT1* knockdown, which may be associated with impaired autophagy ( Figure 10D‒I). Finally, western blot analysis revealed downregulation of PINK1 and Parkin expression in the shANT1 group ( Figure 10J‒L), suggesting the importance of mitophagy in the context of *ANT1* knockdown.

## Discussion

This study revealed pronounced upregulation of ANT1 expression in CRC tissues compared with matched adjacent non-tumor tissues. ANT1 levels in CRC cells (SW480, HCT-116, and SW620) were significantly higher than those in normal human colonic epithelial cells. Chen *et al*. [27] supports our conclusion that ANT1 is indeed expressed at higher levels in CRC tissues than in normal tissues. Similar to the observations of Lan *et al*. [28] in gastric cancer, our study revealed significant upregulation of ANT1 in CRC tissues, suggesting that ANT1 may contribute to a shared pathogenic mechanism in gastrointestinal malignancies. While Chen *et al*. [29] reported only an upregulation at the mRNA level in acute monocytic leukemia, our study in CRC confirmed elevated ANT1 expression at the protein level and revealed its correlation with clinical prognosis. Subsequently, Kaplan-Meier curves were plotted to analyze the associations between ANT1 expression and patient survival and recurrence within a 60-month follow-up period. The results revealed that patients with high ANT1 expression presented significantly shorter survival times and higher recurrence rates, indicating a negative impact of high ANT1 expression on patient prognosis. For advanced-stage tumor patients, studies have indicated a correlation between ANT1 and the cachexia phenomenon [30], which is closely related to the loss of muscle mass. The study revealed significantly higher ANT1 expression in a rabbit model of tumor cachexia than in the control group. These findings suggest that ANT1 may have therapeutic potential for CRC patients who have lost the opportunity for a radical cure. Targeting ANT1 could improve cachexia symptoms and increase the survival time of this subgroup of patients.

ANT1 has been shown to exhibit cytotoxic effects and induce cell death in various cancer cell types, such as cervical cancer, breast cancer, and rhabdomyosarcoma (RMS) [ 15, 31, 32] . However, ANT1 plays a pro-cancer role in ADF human glioblastoma cells, and its downregulation induces oxidative stress and programmed cell death in these cells [33]. Thus, ANT1 exhibits heterogeneity in its physiological functions across different tumor types. In this study, using RNA interference technology, the expression of ANT1 was reduced in highly expressing CRC cell lines (SW480 and SW620), and the effects of *ANT1* knockdown on CRC cell proliferation, migration, and invasion were further analyzed. The results revealed that ANT1 downregulation inhibited malignant biological behaviors such as proliferation, migration, and invasion in CRC cells, suggesting a pro-cancer role of ANT1 in CRC. When cancer cells are highly migratory and invasive, they tend to metastasize more easily [ 34, 35] . Twenty percent of CRC cases at first diagnosis are metastatic CRC, and another 25% of patients with limited disease develop metastasis at a later stage [36]. Metastatic CRC has a poor prognosis, with a 5-year survival rate of less than 20%. Therefore, *ANT1* knockdown can reduce the risk of tumor metastasis, which is essential for controlling CRC progression.

This study revealed that the elevated expression of ANT1 in CRC serves as a driving force in tumorigenesis rather than a secondary consequence. First, ANT1 is markedly overexpressed in multiple CRC cell lines and clinical specimens, and its downregulation significantly curtails malignant phenotypes such as proliferation, migration, and invasion, suggesting a direct role in tumor initiation and progression. Second, ANT1 modulates mitochondrial function by regulating PINK1/Parkin-mediated mitophagy, a process critical to mitochondrial homeostasis, with mitochondrial dysfunction recognized as an early hallmark of cancer [37]. Nevertheless, the possibility that ANT1 overexpression represents an adaptive response to tumor metabolic reprogramming cannot be entirely dismissed. Future investigations employing temporal approaches, such as inducible *ANT1* knockout models, could further elucidate its causal role.

Mitochondrial dysfunction in CRC precipitates oncogenesis and disease progression [38], with ANT1 playing a crucial role in maintaining the normal function of mitochondria [ 39, 40] . Mitochondrial dysfunction can lead to deficient ATP production and abnormal ROS production [41]. It has been established that ANT1 deficiency is linked to increased mitochondrial ROS [ 33, 42] . Excessive ROS can further damage the mitochondrial membrane, alter membrane permeability, and reduce the concentration difference between ions inside and outside the membrane through free diffusion, resulting in a decrease in the membrane potential [ 43– 45] . The loss of ANT1 leads to impaired mitophagy, as evidenced by clinical symptoms and mitochondrial observations in a patient carrying a homozygous loss-of-function mutation in ANT1 [21]. The present study is in line with previous findings that *ANT1* knockdown leads to the accumulation of damaged mitochondria in SW620 cells, enhances ROS generation, decreases the mitochondrial membrane potential, and induces apoptosis. In diverse tumor microenvironments, ANT1 influences mitochondrial function through either its absence or overexpression. For example, in RMS, the loss of ANT1 expression disrupts mitochondrial function, thereby bolstering the oncogenic properties of RMS tumor cells [15]. Conversely, in MDA-MB-231 breast cancer cells, ANT1 transfection induces apoptosis, accompanied by disruption of the mitochondrial membrane potential, consequently suppressing tumor growth *in vivo* [32]. However, these studies did not explore the involvement of mitophagy. In contrast, the present study confirmed that ANT1 overexpression in CRC induces mitochondrial dysfunction, thereby promoting mitophagy. Both *in vitro* and *in vivo*, *ANT1* knockdown significantly inhibited autophagosome induction (downregulation of Beclin-1), autophagosome elongation (downregulation of ATG5 and ATG16L), and autophagosome formation (downregulation of ATG3, ATG7, and LC3-II) in CRC cells; reduced autophagic flux (increased P62); and inhibited ubiquitin-dependent PINK1/Parkin-mediated mitophagy.

The PINK1/Parkin pathway is considered one of the key pathways regulating mitophagy and is involved primarily in the clearance of damaged mitochondria [ 46, 47] . Under normal conditions, PINK1 levels are extremely low; however, when mitochondria become damaged, PINK1 entry into the inner mitochondrial membrane is blocked, leading to PINK1 accumulation on the outer mitochondrial membrane, where it recruits Parkin to damaged mitochondria [48]. PINK1 functions upstream of Parkin, and together, they cooperatively regulate mitophagy. Our experimental findings revealed that *ANT1* knockdown significantly decreased the protein expression levels of PINK1 and Parkin. Although the connection between ANT1 and PINK1/Parkin-mediated mitophagy has been demonstrated in benzo(a)pyrene-induced ovarian toxicity, its role in CRC remains unclear [49] and unconfirmed in tumor-related studies. Our research represents the first demonstration of ANT1’s positive regulatory effect on the PINK1/Parkin pathway in CRC. To investigate the role of the PINK1/Parkin pathway in the anti-CRC function of ANT1, we performed a rescue experiment in which PINK1 was overexpressed. Notably, Parkin overexpression partially reversed the effects of *ANT1* knockdown, including the restoration of proliferation, invasion, and migration capacity suppressed by *ANT1* knockdown; amelioration of mitochondrial function; and inhibition of apoptosis. These findings further suggest that PINK1 may act as a downstream effector of ANT1, counteracting the effects of ANT1 deficiency by restoring mitophagy and mitochondrial function. Thus, targeting PINK1/Parkin-mediated mitophagy through *ANT1* knockdown could be a promising anticancer strategy for CRC treatment. Potential therapeutic approaches may include targeted suppression of PINK1/Parkin expression in CRC or the use of mitophagy inhibitors to exacerbate mitochondrial dysfunction, thereby impeding CRC progression.

While this study preliminarily revealed the potential role of ANT1 in CRC and its underlying mechanisms through various *in vitro* and *in vivo* experiments, several limitations remain. First, our experiments focused primarily on SW620 and SW480 cell lines, and further studies are needed to verify whether these findings are broadly applicable to other CRC cell lines or even other tumor types. This study did not assess the microsatellite instability (MSI) status of patients, which may represent a confounding factor given the distinct molecular profiles of MSI-high and microsatellite-stable (MSS) colorectal cancers. Future studies should evaluate whether ANT1’s role varies by MS status. Additionally, although we confirmed the inhibitory effect of ANT1 on tumor growth *in vivo* via a mouse xenograft model, further validation in clinical samples, such as through larger patient cohorts and multicenter studies, is an important next step. Future research should expand to more complex models and further investigate the potential of ANT1 and its associated molecular pathways as therapeutic targets in CRC treatment.

In conclusion, our study demonstrated that *ANT1* knockdown inhibited PINK1/Parkin-mediated mitophagy, thereby effectively suppressing the progression of CRC. These findings reveal the important role of ANT1 in CRC and provide a theoretical basis for further investigations of the potential of ANT1 as a therapeutic target. These findings may have significant clinical implications for the treatment and prevention of CRC.
